# Metabolic Profile of Skimmianine in Rats Determined by Ultra-Performance Liquid Chromatography Coupled with Quadrupole Time-of-Flight Tandem Mass Spectrometry

**DOI:** 10.3390/molecules22040489

**Published:** 2017-03-23

**Authors:** Aihua Huang, Hui Xu, Ruoting Zhan, Weiwen Chen, Jiawei Liu, Yuguang Chi, Daidi Chen, Xiaoyu Ji, Chaoquan Luo

**Affiliations:** 1 Key Laboratory of Ministry of Education, Research Center of Chinese Herbal Resources and Engineering, Guangzhou University of Chinese Medicine, Guangzhou 510006, China; hah2008@gzucm.edu.cn (A.H.); zhanrt@gzucm.edu.cn (R.Z.); chenww@gzucm.edu.cn (W.C.); liujw@gzucm.edu.cn (J.L.); jixy2017@163.com (X.J.); 18825146206@163.com (C.L.); 2 School of Chinese Materia Medica, Guangzhou University of Chinese Medicine, Guangzhou 510006, China; ygchi@gzucm.edu.cn (Y.C.); 13434325639@163.com (D.C.)

**Keywords:** skimmianine, metabolites, UPLC/Q-TOF-MS, furoquinoline

## Abstract

Skimmianine is a furoquinoline alkaloid present mainly in the Rutaceae family. It has been reported to have analgesic, antispastic, sedative, anti-inflammatory, and other pharmacologic activities. Despite its critical pharmacological function, its metabolite profiling is still unclear. In this study, the in vivo metabolite profiling of skimmianine in rats was investigated using ultra-performance liquid chromatography coupled with quadrupole time-of-flight tandem mass spectrometry (UPLC/Q-TOF-MS). The metabolites were predicted using MetabolitePilot^TM^ software. These predicted metabolites were further analyzed by MS^2^ spectra, and compared with the detailed fragmentation pathway of the skimmianine standard and literature data. A total of 16 metabolites were identified for the first time in rat plasma, urine, and feces samples after oral administration of skimmianine. Skimmianine underwent extensive Phase I and Phase II metabolism in rats. The Phase I biotransformations of skimmianine consist of epoxidation of olefin on its furan ring (M1) followed by the hydrolysis of the epoxide ring (M4), hydroxylation (M2, M3), *O*-demethylation (M5-M7), didemethylation (M14–M16). The Phase II biotransformations include glucuronide conjugation (M8–M10) and sulfate conjugation (M11–M13). The epoxidation of 2,3-olefinic bond followed by the hydrolysis of the epoxide ring and *O*-demethylation were the major metabolic pathways of skimmianine. The results provide key information for understanding the biotransformation processes of skimmianine and the related furoquinoline alkaloids.

## 1. Introduction

Skimmianine is a natural furoquinoline alkaloid widely present in plants of Rutaceae family, including *Rutagravelens* L. [1], *Zanthoxylum nitidum* [2], *Zanthoxylum simulans* Hance [3], and *Dictamnus dasycarpus* [4] etc. Skimmianine possesses non-narcotic analgesic [5], antispastic, sedative [3], antibacterial [6], antivirus [7], and other pharmacological activities, as well as antiplatelet aggregation [8]. It has been proposed to act as a potential treatment for Alzheimer’s disease owing to its function of inhibiting acetylcholine esterase [2] and nitric oxide (NO) production [9]. Recently, skimmianine has been shown to exhibit an anti-inflammatory effect on ear and paw edema models. The mechanisms of its action included the suppression of *TNF*-α and *IL*-6 gene transcription, the inhibition of the production of NO, prostaglandin E2, and superoxide anions, as well as elastase-release [1,10,11]. Furthermore, skimmianine also exhibited cytotoxicity against a variety of cancer cell lines [12,13,14,15] and genotoxicity [16,17].

As is well known, drug metabolism is closely related to drug bioactivities and toxicity; therefore, identification of drug metabolites is of vital importance for elucidating drug pharmacological mechanisms and pharmacokinetic behavior. Limited information is known about the metabolism and pharmacokinetics of skimmianine in spite of our deep understanding of its pharmacological activities and toxicity. Available studies indicate that skimmianine has been distributed and eliminated rapidly after intravenous administration [18] and that it has been completely absorbed across intestinal epithelial cells [19]. No studies on the metabolites produced in vitro or in vivo have been reported. Nowadays, ultra-performance liquid chromatography coupled with quadrupole time-of-flight tandem mass spectrometry (UPLC/Q-TOF-MS) has become a valuable tool for drug metabolite identification due to its selectivity, sensitivity, and speediness [20]. In this paper, the method of UPLC/Q-TOF-MS was developed for the characterization of metabolic profiles in rat plasma, urine, and feces after oral administration of skimmianine. The results would provide an important basis for further pharmacological studies and biosafety evaluation.

## 2. Results and Discussion

### 2.1. Mass Spectral Properities of Skimmianine

**Figure 1:**
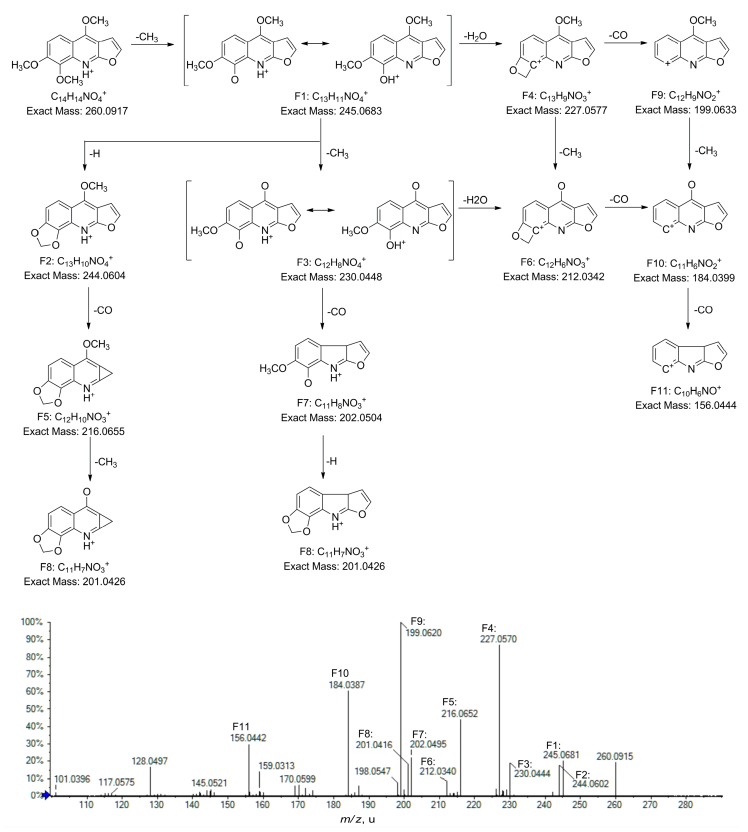
MS^2^ spectrum and the proposed fragmentation pathways of skimmianine standard.

To identify the metabolites of skimmianine in rats, the skimmianine standard was firstly analyzed by UPLC/Q-TOF-MS under the optimized conditions. The chromatogram and MS^2^ spectra were recorded and the fragmentation pathways were proposed (Figure 1 and Figure S1).

Skimmianine presented parent ion at *m/z* 260.0917 (C_14_H_14_NO_4_^+^) at 15.5 min. The fragment ion at *m/z* 245.0681 (C_13_H_11_NO_4_^+^, F1) was attributed to the elimination of CH_3_. It may undergo an intramolecular proton transfer from NH^+^ to C8-OH^+^. The subsequent loss of H_2_O of F1 resulted in a fragment ion at *m/z* 227.0575 (C_13_H_9_NO_3_^+^, F4). The successive loss of H_2_O following CH_3_ might be a typical characteristic of skimmianine due to its vicinal methoxy groups at C7 and C8. F4 further fragmented in two ways, generating fragment ions at *m/z* 199.0633 (C_12_H_9_NO_2_^+^, F9), 212.0342 (C_12_H_6_NO_3_^+^, F6), and 184.0399 (C_11_H_6_NO_2_^+^, F10). While the loss of CH_3_ following CO led to the formation of F9 and F10, F6 and F10 were produced vice versa. The ion at *m/z* 156.0444 (C_10_H_6_NO^+^, F11) was produced by the neutral loss of CO from F10. F4, F9, and F10 were the dominant peaks in the MS^2^ spectrum of skimmianine, implying that the successive elimination of CH_3_ and H_2_O must be the major fragmentation pathway. The minor peaks observed in the MS^2^ spectrum should be a result of other pathways. F1 might break up into the ions at *m/z* 244.0602 (C_13_H_10_NO_4_^+^, F2), 216.0652 (C_12_H_10_NO_3_^+^, F5), and 201.0426 (C_11_H_7_NO_3_^+^, F8) after the consecutive loss of H, CO, and CH_3_. Otherwise, F1 might undergo the successive elimination of CH_3_, CO, and H, producing the ions at *m/z* 230.0448 (C_12_H_8_NO_4_^+^, F3), 202.0504 (C_11_H_8_NO_3_^+^, F7), and 201.0426 (C_11_H_7_NO_3_^+^, F8).

### 2.2. Structure Elucidation of Skimmianine Metabolites

**Figure 2:**
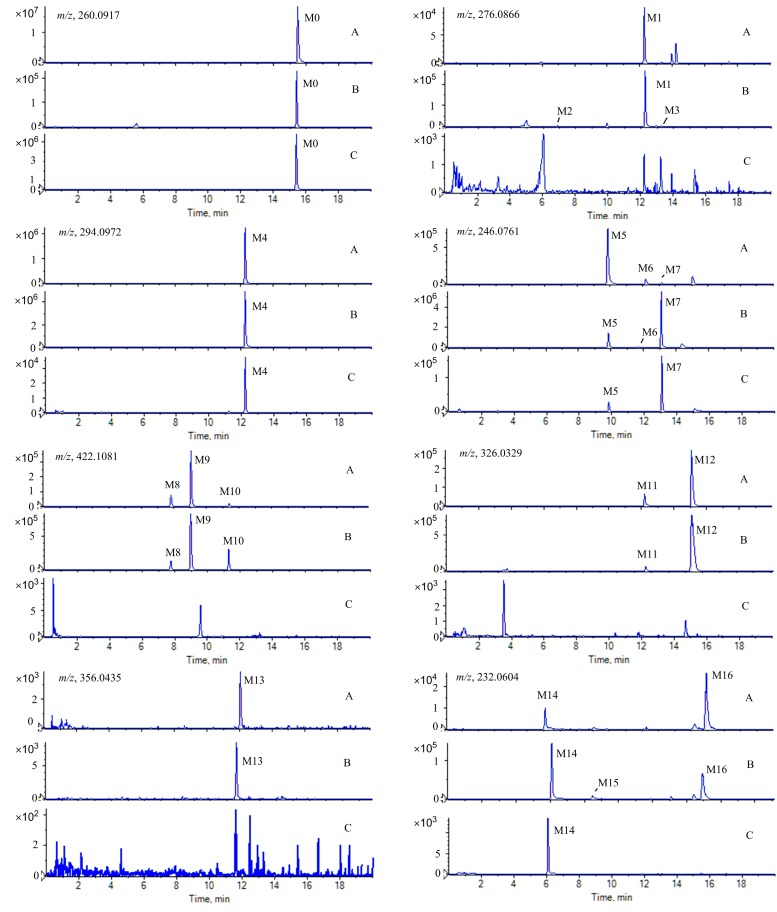
Extracted ion chromatograms of skimmianine and its metabolites in rats: A, plasma samples; B, urine samples; C, feces samples.

After the oral administration of skimmianine to rats, plasma, urine, and feces samples were collected, extracted, and analyzed as described. The metabolites were predicted by using MetabolitePilot^TM^ software and then confirmed manually based on the above fragmentation pathways of the skimmianine standard and the literature [21,22,23]. A total of 16 metabolites were identified in the biological samples originating from rats (Table 1). Extract ion chromatograms (EICs) of skimmianine and its metabolites are shown in Figure 2, and the related EICs in blank plasma, urine, and feces are shown in Figure S2 (see supplementary).

M1, M2, and M3 with protonated molecular weights of 276.0864, 276.0874, and 276.0861, respectively, were predicted to have the same molecular formula (C_14_H_14_NO_5_^+^). These three metabolites harbored one additional O atom compared to skimmianine, indicating that they all resulted from an addition reaction of oxygen to skimmianine. M1 was eluted at 12.3 min and generated a fragment ion at *m/z* 248.0918 (C_13_H_14_NO_4_^+^) through the neutral loss of CO from the parent ion, suggesting that the O atom must be added to the C2–C3 double bond to form an epoxide. Subsequently, the fragment ions at *m/z* 233.0679 (C_12_H_11_NO_4_^+^), 232.0608 (C_12_H_10_NO_4_^+^), and 204.0655 (C_11_H_10_NO_3_^+^) were produced via the continuous loss of CH_3_, H, and CO. The fragment ion at *m/z* 233.0679 (C_12_H_11_NO_4_^+^) might have another fragmentation route, producing fragment ions at *m/z* 205.0613 (C_11_H_11_NO_3_^+^), 190.0834 (C_1__0_H_8_NO_3_^+^), 175.0618 (C_9_H_5_NO_3_^+^), and 147.0674 (C_8_H_5_NO_2_^+^) as a result of the consecutive loss of CO, CH_3_, CH_3_, and CO. The fragment ion at *m/z* 162.0909 (C_9_H_8_NO_2_^+^) was produced by the elimination of CO from the ion at *m/z* 190.0834 (C_1__0_H_8_NO_3_^+^). From the ion at *m/z* 248.0918 (C_13_H_14_NO_4_^+^), another fragmentation route produced ions at *m/z* 217.0735 (C_12_H_11_NO_3_^+^), 216.0659 (C_12_H_10_NO_3_^+^), and 188.0708 (C_11_H_10_NO_2_^+^) because of the consecutive loss of CH_3_O, H, and CO. Based on the above fragmentation details, M1 was identified as a 2,3-epoxide metabolite of skimmianine.

M2 and M3 at retention times of 6.9 and 13.3 min, respectively, yielded fragment ions at *m/z* 261.0627 (C_13_H_11_NO_5_^+^), 243.0527 (C_13_H_9_NO_4_^+^), 215.0565 (C_1__2_H_9_NO_3_^+^), and 200.0333 (C_11_H_6_NO_3_^+^). These ions were all 16 u more than those of skimmianine, suggesting that the fragmentation pathway was in complete accordance with that of the parent drug. M2 and M3 were thus identified as C5-hydroxylation and C6-hydroxylation metabolites of skimmianine, respectively.

M4 with *m/z* 294.0972 (C_14_H_16_NO_6_^+^) was 34 u more than skimmianine, indicating that it was an oxidation and internal hydrolysis product of skimmianine. It produced fragment ions at *m/z* 276.0862 (C_14_H_14_NO_5_^+^), 248.0918 (C_13_H_14_NO_4_^+^), 217.0735 (C_12_H_11_NO_3_^+^), 216.0659 (C_12_H_10_NO_3_^+^), 188.0708 (C_11_H_10_NO_2_^+^), and 175.0618 (C_9_H_5_NO_3_^+^). These ions were identical to those of M1. Therefore, M4 was identified as 2,3-epoxide hydrolysis product of M1.

M5, M6, and M7 at rentention times of 9.9, 11.7, and 13.1 min shared the same formula (C_13_H_12_NO_4_^+^). These metabolites lost one C and two H atoms from skimmianine, indicating that these metabolites were demethylation products of skimmianine. M5 along with its fragment ions at *m/z* 231.0531 (C_12_H_9_NO_4_^+^), 213.0419 (C_12_H_7_NO_3_^+^), and 185.0470 (C_11_H_7_NO_2_^+^) were all 14 u less than those of skimmianine. The fragmentation pattern was consistent with that of the parent molecule. M5 was thus identified as C4-*O*-demethylation product of skimmianine. In the case of M6 and M7, the fragmentation pattern was different from that of skimmianine due to the lack of vicinal methoxy groups at C7 and C8. The fragment ions at *m/z* 231.0526 (C_12_H_9_NO_4_^+^), 216.0289 (C_11_H_6_NO_4_^+^), 188.0327 (C_10_H_6_NO_3_^+^), and 160.0384 (C_9_H_6_NO_2_^+^) were produced owing to the consecutive loss of CH_3_, CH_3_, CO, and CO. Because C7-OH is less polar than C8-OH, M6 and M7 were identified as C8-*O*-demethylation and C7-*O*-demethylation products of skimmianine, respectively.

Eluted at 7.7, 8.9, and 11.3 min, respectively, M8, M9, and M10 were deduced to have the same formula (C_19_H_20_NO_10_^+^). All these metabolites displayed a neutral loss of 176 u, suggesting that they were demethylated and glucuronidated products of skimmianine. M8 yielded fragment ions at *m/z* 246.0759 (C_13_H_12_NO_4_^+^), 231.0525 (C_12_H_9_NO_4_^+^), 213.0425 (C_12_H_7_NO_3_^+^), and 185.0471 (C_11_H_7_NO_2_^+^) via the sequential loss of C_6_H_8_O_6_, CH_3_, H_2_O, and CO. The dominant fragment ions were similar to those of M5. M8 was identified as a glucuronidated product of M5. Both M9 and M10 yielded fragment ions at *m/z* 246.0759 (C_13_H_12_NO_4_^+^), 231.0525 (C_12_H_9_NO_4_^+^), 216.0289 (C_11_H_6_NO_4_^+^), and 188.0339 (C_1__0_H_6_NO_3_^+^). The fragmentation pattern was consistent with that of M6 and M7. As the polarity of M9 is larger than that of M10, M9 and M10 were assigned to glucuronidated products of M6 and M7, respectively.

M11 and M12 had the same formula (C_13_H_12_NO_7_S^+^), and these two metabolites were eluted at 11.7 and 14.4 min, respectively. Both metabolites displayed a neutral loss of 80 u, suggesting that they were demethylated and sulfated products of skimmianine. M11 and M12 also yielded fragment ions at *m/z* 246.0759 (C_13_H_12_NO_4_^+^), 231.0525 (C_12_H_9_NO_4_^+^), 216.0289(C_11_H_6_NO_4_^+^), and 188.0339 (C_1__0_H_6_NO_3_^+^). This was also consistent with that of M6 and M7. M11 and M12 were identified as sulfated products of M6 and M7, respectively.

M13 displayed a protonated molecular ion at *m/z* 356.0435 (C_14_H_14_NO_8_S^+^) at retention time 12.0 min. It further yielded a fragment ion at *m/z* 276.0866 (C_14_H_14_NO_5_^+^) via a neutral loss of 80 u, suggesting that M13 was an oxygenated and sulfated product of skimmianine. The fragment ions were similar to those of M2 and M3. Moreover, considering the elution time of these metabolites, M13 was identified as the sulfated product of M3.

M14, M15, and M16 were eluted at 6.1, 8.5, and 14.9 min, respectively. They were supposed to share the same molecular formula (C_12_H_10_NO_4_^+^), C_2_H_4_ less than the parent molecule. All of these metabolites generated fragment ions at *m/z* 217.0371 (C_1__1_H_7_NO_4_^+^), 189.0427 (C_1__0_H_7_NO_3_^+^), 161.0468 (C_9_H_7_NO_2_^+^), and 133.0522 (C_8_H_7_NO^+^) via the continuous loss of CH_3_, CO, CO, and CO. According to the polarity of molecules, they were speculated as C4, C8-*O*-didemethylation; C4, C7-*O*-didemethylation and C7, C8-*O*-didemethylation products of skimmianine.

**Figure 3:**
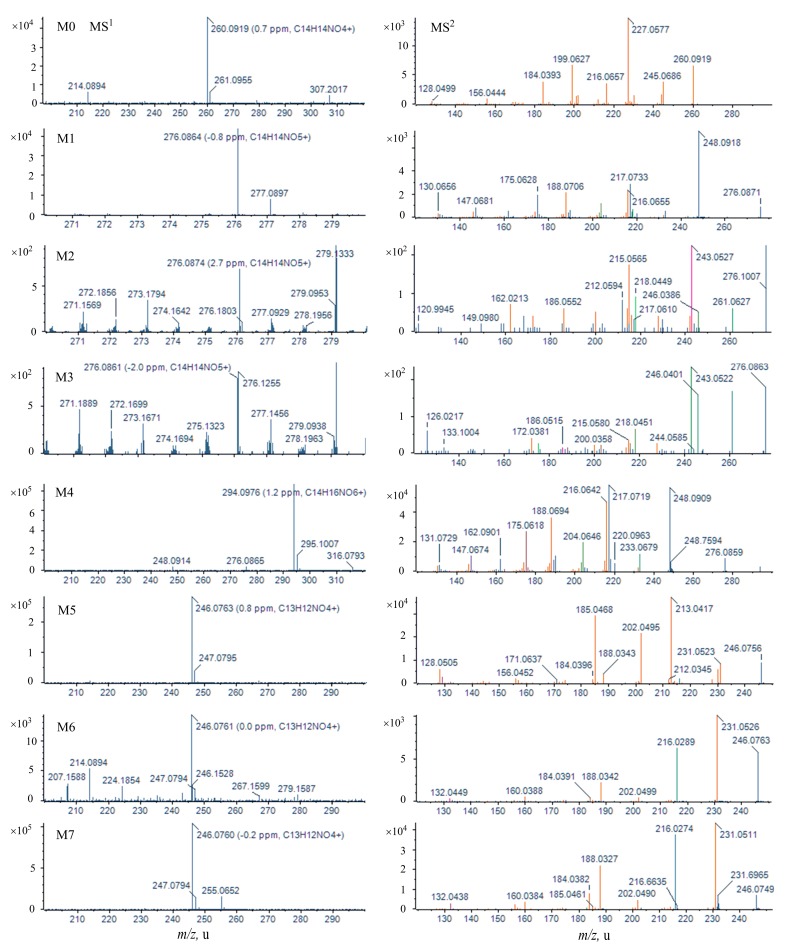

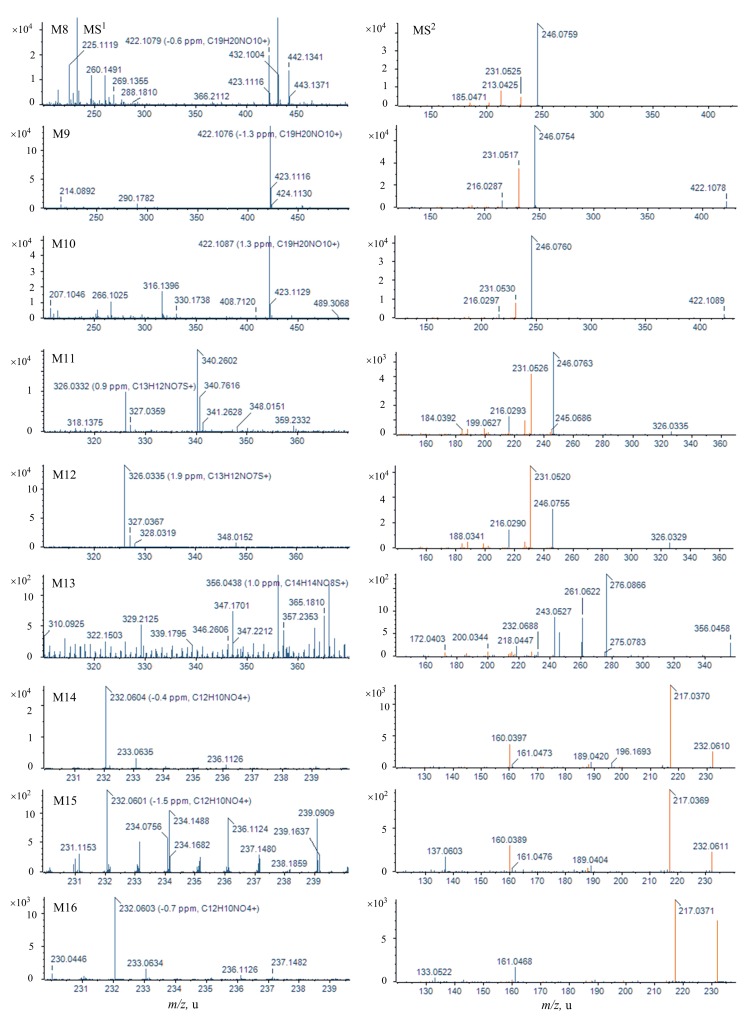
MS^1^ and MS^2^ spectra of the parent drug and its metabolites identified in rat urine sample.

**Figure 4:**
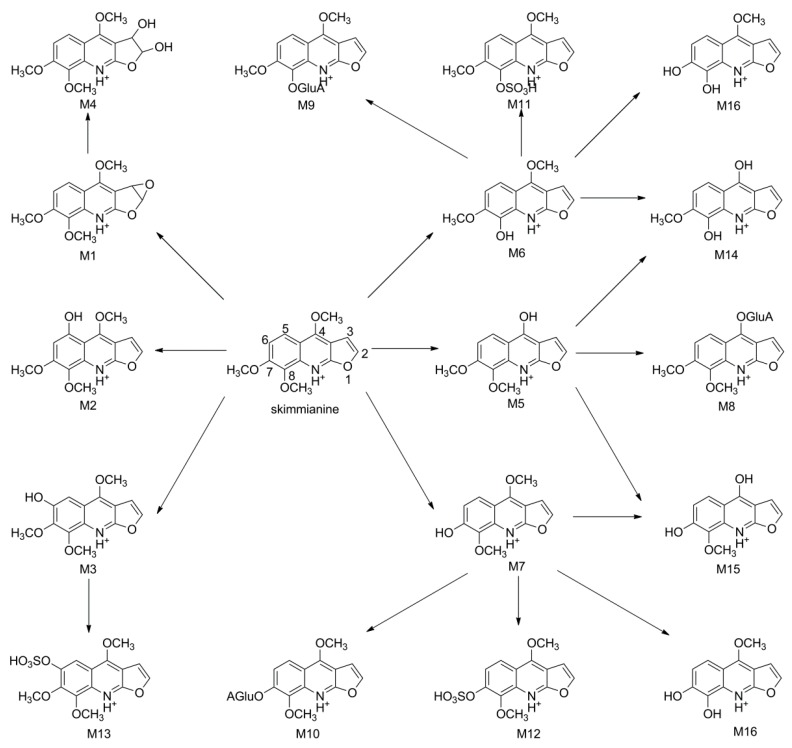
Proposed metabolic pathways of skimmianine in rats.

Figure 3 showed MS^1^ and MS^2^ spectra of the parent drug and its metabolites identified in rat urine samples. The mass spectra of metabolites in plasma and feces were listed in Figures S3 and S4. The proposed chemical structures and metabolic pathways of skimmianine metabolites are summarized in Figure 4. Since all metabolites were identified using high-resolution mass spectrometry, further studies are needed to confirm their structures and configurations.

### 2.3. Metabolic Profile of Skimmianinein Rats

Skimmianine was administered to rats orally and the structures of its 16 metabolites were identified. The metabolites included 10 Phase I compounds (M1–7, M14–16) and 6 Phase II ones (M8–13). The Phase I metabolism was composed of *O*-demethylation, didemethylation, hydroxylation, and epoxidation of olefin on the furan ring of skimmianine and followed by the hydrolysis of its epoxide ring. The Phase II metabolism included glucuronidation and sulfation of some Phase I metabolites. The Phase I metabolic pathways were similar to those of dictamnine, which was also a furoquinoline alkaloid [21,22,23]. It seemed that the epoxidation of 2,3-olefinic on the furan ring followed by the hydrolysis of its epoxide ring was a typical characteristic of the furoquinoline alkaloid metabolism. Besides that, *O*-demethylation of the methoxy group must also be a major pattern in the Phase I metabolism. In comparison, metabolites resulting from the oxidation of the benzene moiety were merely detectable. The major pathway of the Phase II metabolism was glucuronidation and sulfation after *O*-demethylation.

As shown in Figure 2, the distribution of the metabolites was tissue-specific. While all 16 (Figure 3) metabolites were found in rat urine, 13 (Figure S3) and 4 (Figure S4) metabolites were detected in plasma and feces, respectively. Four metabolites (M4, M5, M7, and M14) were common in the rat plasma, urine, and feces (Figure 2). M4 was observed as the most abundant metabolite of skimmianine in plasma and urine. Comparing the metabolic profile in urine with that in plasma, the level of skimmianine was observed to have decreased dramatically, suggesting that skimmianine was widely metabolized in rats and excreted mainly through urine in the form of metabolites.

Various biological activities and genotoxicity of skimmianine might correlate with its strong metabolism. It has been reported that 8-hydroxydictamnine, a hydroxylation product of dictaminine, showed strong mutagenicity with liver extracts of Wistar rats injected with phenobarbital, but 7-hydroxydictamnine and other furoquinolines with a hydroxy group at C7 were inactive in the *Salmonella typhimurium* strain TA98 [23]. In this study, M6 possessed a hydroxy group at C8 and was observed in plasma and urine. Whether M6 is related to mutagenicity needs to be determined with further investigation. An assessment of the contribution to the overall activity or toxicity from key metabolites would be interesting.

## 3. Materials and Methods

### 3.1. Chemical, Reagents, and Materials

Skimmianine (No. 160106, purity 98.0%) was purchased from Shanghai Yuan-Mu Bio-Technology Ltd. Co., (Shanghai, China). Acetonitrile and formic acid of HPLC grade agents were purchased from Merck (Darmstadt, Germany). All other agents were of analytical grade and obtained from Guangzhou Chemical Reagent Factory (Guangzhou, China). Purified water was prepared from a Milli-Q system (Millipore Billerica, MA, USA). The HyperSep C_18_ solid-phase extraction (SPE) column (1000 mg, 6 mL) was purchased from Thermo Electron Corporation (Waltham, MA, USA).

### 3.2. Instrumentation and Analytical Conditions

Chromatographic separation was performed on a Shimadzu Nexera UHPLC LC-30A system (Shimadzu Corporation, Tokyo, Japan) with an ODS column (Shimadzu, 2.0 mm i.d. × 100 mm) at 35 °C. The mobile phase, consisting of Solvent A (0.1% formic acid solution) and Solvent B (0.1% formic acid acetonitrile), was delivered at a flow rate of 0.4 mL/min using a gradient program as follows: 0–10 min, 5%–20% B; 10–15 min, 20%–35% B; 15–20 min, 35%–100% B. Each injection volume was 3 μL.

MS data were acquired on an AB SCIEX Triple TOF 5600 (AB Sciex Pte. Ltd., Singapore). The system was controlled with AB SCIEX Analyst TF (Version 1.7) software (AB Sciex Pte. Ltd., Singapore, Singapore). Information-dependent acquisition (IDA) and mass defect filter information-dependent acquisition (MDF-IDA) were used for identifying metabolites. Mass spectrometric conditions were as follows: interface, positive electrospray ionization (ESI); gas 1 and 2, nitrogen 50 psi; curtain gas, nitrogen 40 psi; source temperature 500 °C; ion spray voltage 5500 V; declustering potential 100 V, scan range 100–800 Da, and collision energy 10 eV.

### 3.3. Animals, Dosage and Biological Sample Collection

Male Sprague–Dawley rats (250 ± 20 g) used in this study were provided by the Experimental Animal Center of Guangzhou University of Chinese Medicine. The laboratory animal license number is SCXK 2013-0020. These animals were maintained in an air-conditioned animal facility at 23 ± 2 °C, with a humidity of 55% ± 5% and a 12 h light/dark cycle for 5 days before use. The rats had free access to water and a standard diet. Animal welfare and experimental procedures were strictly in accordance with the guidelines of the Committee on the Care and Use of Laboratory Animals in China and the related ethical regulations of Guangzhou University of Chinese Medicine.

The rats were randomly divided into 3 groups, with A for the blank control group, B for plasma collection group, and C for the urine and feces collection group, 6 rats per group. Before administration, the rats were fasted for 12 h but were allowed water access ad libitum. Skimmianine suspension in a 0.5% carboxymethyl cellulose sodium aqueous solution was orally administered to Groups B and C at a dose of 20 mg·kg^−1^ body weight, while a 0.5% carboxymethyl cellulose sodium aqueous solution was orally administered to Group A. 

The rats of Group B were anesthetized at 0.5 h, 1 h, 2 h, 3 h, 4 h, and 6 h after doses, respectively. The blood samples were collected from aorta abdominalis in heparinized tubes. All blood samples were then centrifuged at 1274× *g* for 15 min at 4 °C and mixed together to produce the pooled plasma. Blank plasma samples collected from Group A were prepared following the same procedures.

For the collection of urine and feces samples after dose, the rats of Group C were put into metabolic cages individually. The samples were collected at 24 h post-intake, respectively, while control urine and feces samples were collected before drug administration. All samples were stored at −80 °C. The frozen urine and feces samples were thawed at room temperature before use.

### 3.4. Biological Sample Preparation

Two milliliters of plasma sample were loaded onto a pretreated solid-phase extraction (SPE) column. The column was washed with 6 mL of water, followed by elution using 6 mL of methanol. The methanol eluate was evaporated to dryness at 35 °C. The residue was reconstituted in 300 μL of acetonitrile and water (50:50, *v*/*v*) and centrifuged at 15,493× *g* for 15 min. The supernatant was used for LC-MS analysis.

Two milliliters of urine sample were prepared using the same method as the plasma samples with a minor change. Rather than 300 μL of acetonitrile and water (50:50, *v*/*v*), the residue was dissolved in 600 μL of acetonitrile and water (50:50, *v*/*v*) and centrifuged at 15,493× *g* for 15 min. The supernatant was collected for LC-MS analysis.

The feces sample was extracted with 15 times the amount of methanol–water (70:30, *v*/*v*). The extract was centrifuged at 15,493× *g* for 15 min. The supernatant was collected.

### 3.5. Data Processing

Data processing was carried out with Peakview (Version 2.0, AB SCIEX) and MetabolitePilot^TM^ (Version 1.5, AB SCIEX). Possible biotransformations which include hydroxylation, demethylation, ketonization, glucuronidation, sulfate conjugation, and combinations were set to analyze metabolites using MetabolitePilot^TM^ software by comparing the samples and the control.

## 4. Conclusions

In this study, the metabolic profile of skimmianine in rats was characterized based on ultra-performance liquid chromatography coupled with quadrupole time-of-flight tandem mass spectrometry. A total of 16 metabolites of skimmianine were identified tentatively in the rat plasma, urine, and feces after the oral administration of skimmianine. All of these metabolites are reported for the first time in this publication. The predominant metabolic pathways were an epoxidation of 2,3-olefinic on its furan ring and followed by the hydrolysis of its epoxide ring, *O*-demethylation, and subsequent glucuronidation or sulfation. The 2,3-olefinic on the furan ring, the methoxy group, and the H of the benzene ring were the metabolic reactive sites. The results are important for understanding the biotransformation processes of skimmianine and the related furoquinoline alkaloids and provide a basis for the further study of skimmianine.

## Figures and Tables

**Table 1:** MS data and identification results of skimmianine metabolites that appeared in rat plasma, urine, and feces.

NO.	RT (min)	Mass Found	Error (ppm)	Formula [M + H]^+^	MS^2^ Ions	Source	Metabolite Name
M0	15.5	260.0919	0.7	C_14_H_14_NO_4_	245.0681, 244.0602, 227.0575, 216.0652, 199.0633, 184.0399	P U F	Parent
M1	12.3	276.0864	−0.8	C_14_H_14_NO_5_	248.0918, 233.0679, 232.0608, 217.0735, 216.0659, 204.0655, 188.0708	P U	2,3-epoxide of skimmianine
M2	6.9	276.0874	2.7	C_14_H_14_NO_5_	261.0627, 243.0527, 215.0565, 200.0333	U	C5-hydroxylation of skimmianine
M3	13.3	276.0861	−2.0	C_14_H_14_NO_5_	261.0627, 243.0527, 215.0565, 200.0333	U	C6-hydroxylation of skimmianine
M4	12.3	294.0976	1.2	C_14_H_16_NO_6_	276.0862, 248.0918, 217.0735, 216.0659, 188.0708, 175.0618	P U F	2,3-epoxide hydrolysis of M1
M5	9.9,	246.0763	0.8	C_13_H_12_NO_4_	231.0531, 213.0419, 185.0470	P U F	C4-*O*-demethylation of skimmianine
M6	11.7	246.0761	0	C_13_H_12_NO_4_	231.0526, 216.0289, 188.0342, 160.0388	P U	C8-*O*-demethylation of skimmianine
M7	13.1	246.0760	−0.2	C_13_H_12_NO_4_	231.0511, 216.0274, 188.0327, 160.0384	P U F	C7-*O*-demethylation of skimmianine
M8	7.7	422.1079	−0.6	C_19_H_20_NO_10_	246.0759, 231.0525, 213.0425, 185.0471	P U	Glucuronidation of M5
M9	8.9	422.1076	−1.3	C_19_H_20_NO_10_	246.0759, 231.0525, 216.0289, 188.0339	P U	Glucuronidation of M6
M10	11.3	422.1087	1.3	C_19_H_20_NO_10_	246.0760, 231.0530, 216.0297	P U	Glucuronidation of M7
M11	11.7	326.0332	0.9	C_13_H_12_NO_7_S	246.0759, 231.0525, 216.0289, 188.0339	P U	Sulfation of M6
M12	14.4	326.0335	1.9	C_13_H_12_NO_7_S	246.0759, 231.0525, 216.0289, 188.0339	P U	Sulfation of M7
M13	12.0	356.0438	1.0	C_14_H_14_NO_8_S	276.0866, 261.0622, 243.0527, 200.0344	P U	Sulfation of M3
M14	6.1	232.0604	−0.4	C_12_H_10_NO_4_	217.0370, 189.0420, 161.0473	P U F	C4, C8-*O*-Didemethylation of skimmianine
M15	8.5	232.0601	−1.5	C_12_H_10_NO_4_	217.0369, 189.0404, 161.0476	U	C4, C7-*O*-Didemethylation of skimmianine
M16	14.9	232.0603	−0.7	C_12_H_10_NO_4_	217.0371, 189.0427, 161.0468	P U	C7, C8-*O*-Didemethylation of skimmianine

P: plasma; U: urine; F: feces.

## Supplementary Materials

Supplementary materials are available online. Figure S1: Extracted ion chromatogram of skimmianine standard. Figure S2: Extracted ion chromatograms of skimmianine and its metabolites in rats: A, blank plasma sample; B, blank urine sample; C, blank feces sample. Figure S3: MS^1^ and MS^2^ spectra of parent drug and its metabolites identified in rat plasma sample. Figure S4: MS^1^ and MS^2^ spectra of parent drug and its metabolites identified in rat feces sample.
