# Supplementary material for: Metabolic Profile of Skimmianine in Rats Determined by Ultra-Performance Liquid Chromatography Coupled with Quadrupole Time-of-Flight Tandem Mass Spectrometry
Source: Molecules. 2017 Mar 23;22(4):489. doi: 10.3390/molecules22040489 (PMC6154341; doi:10.3390/molecules22040489)
Supplement: Supplementary file 1 [file molecules-22-00489-s001.pdf]

Supplementary material

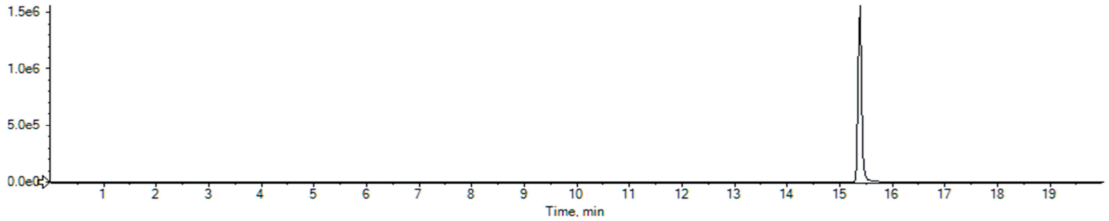

Figure S1. Extracted ion chromatograms of skimmianine standard

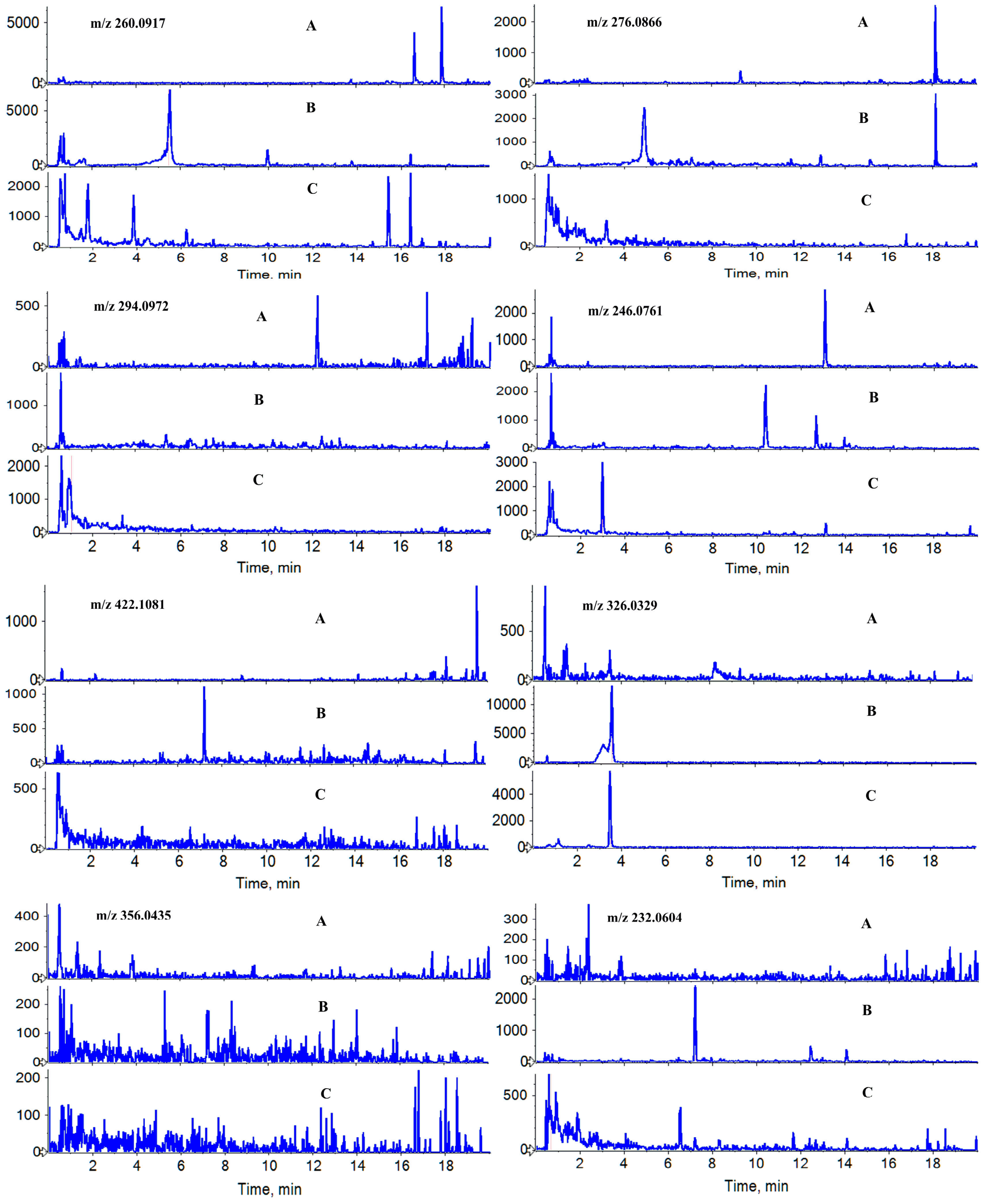

Figure S2. Extracted ion chromatograms of skimmianine and its metabolites in rats: A, blank plasma sample; B, blank urine sample; C, blank feces sample.

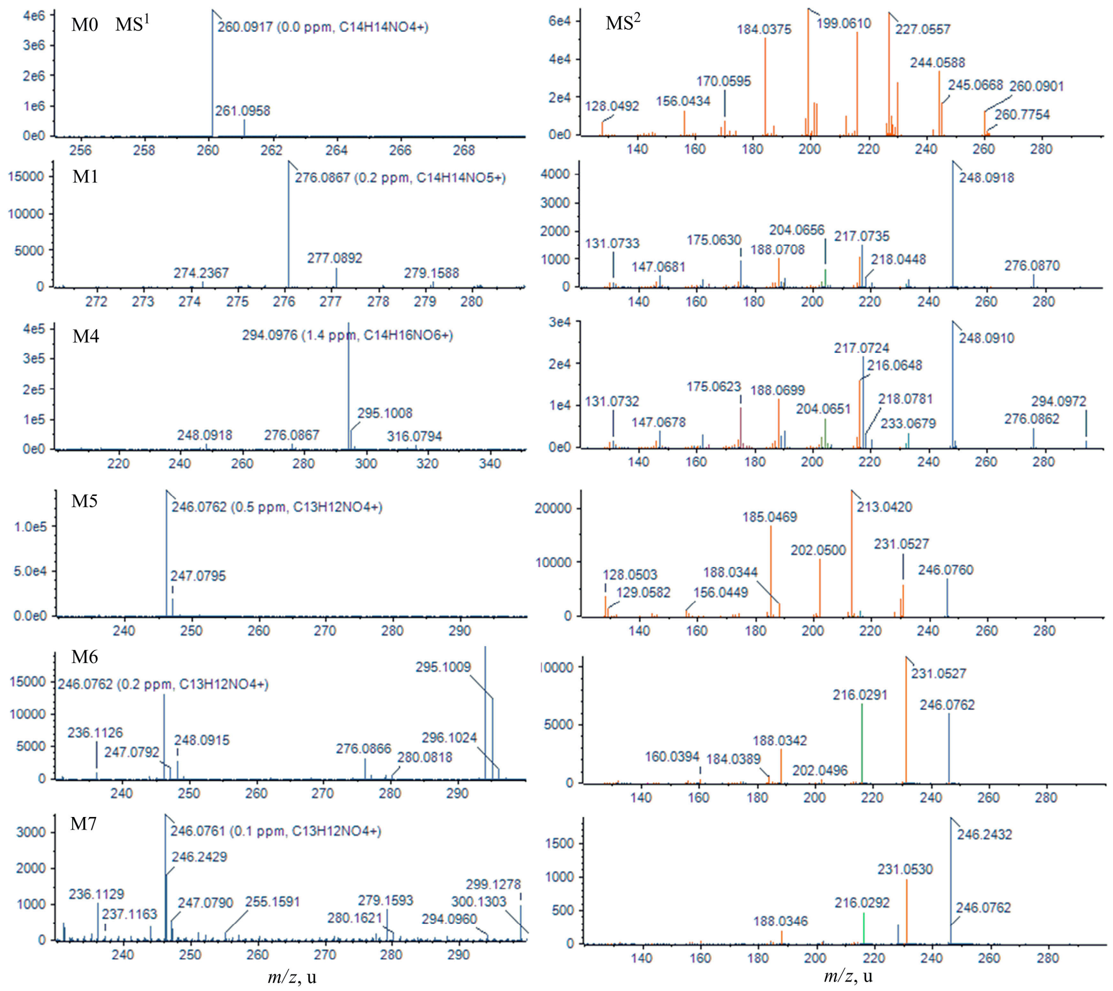

Figure S3. MS¹ and MS² spectra of parental drug and its metabolites identified in rat plasma samples.

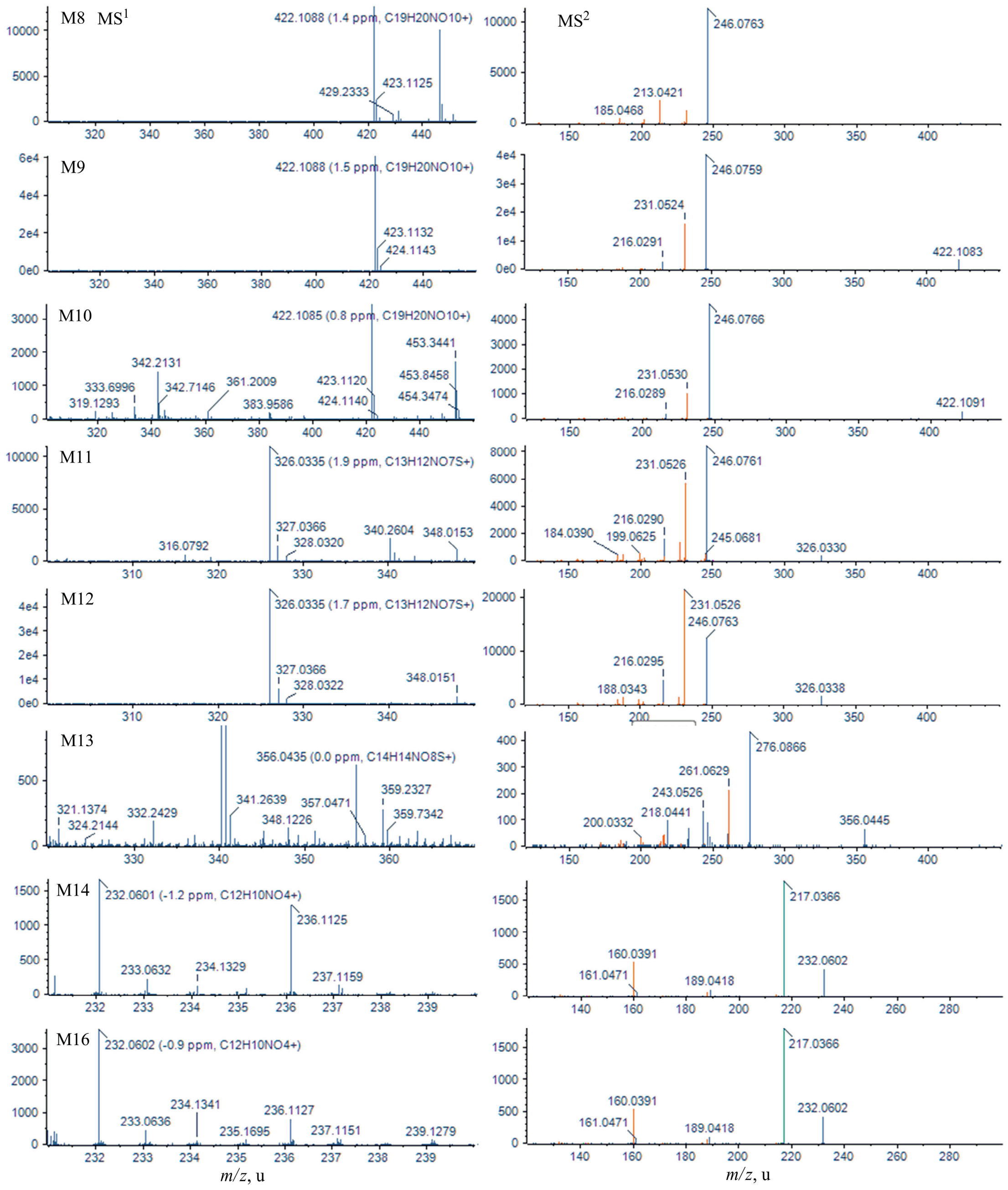

Figure S3. Continued

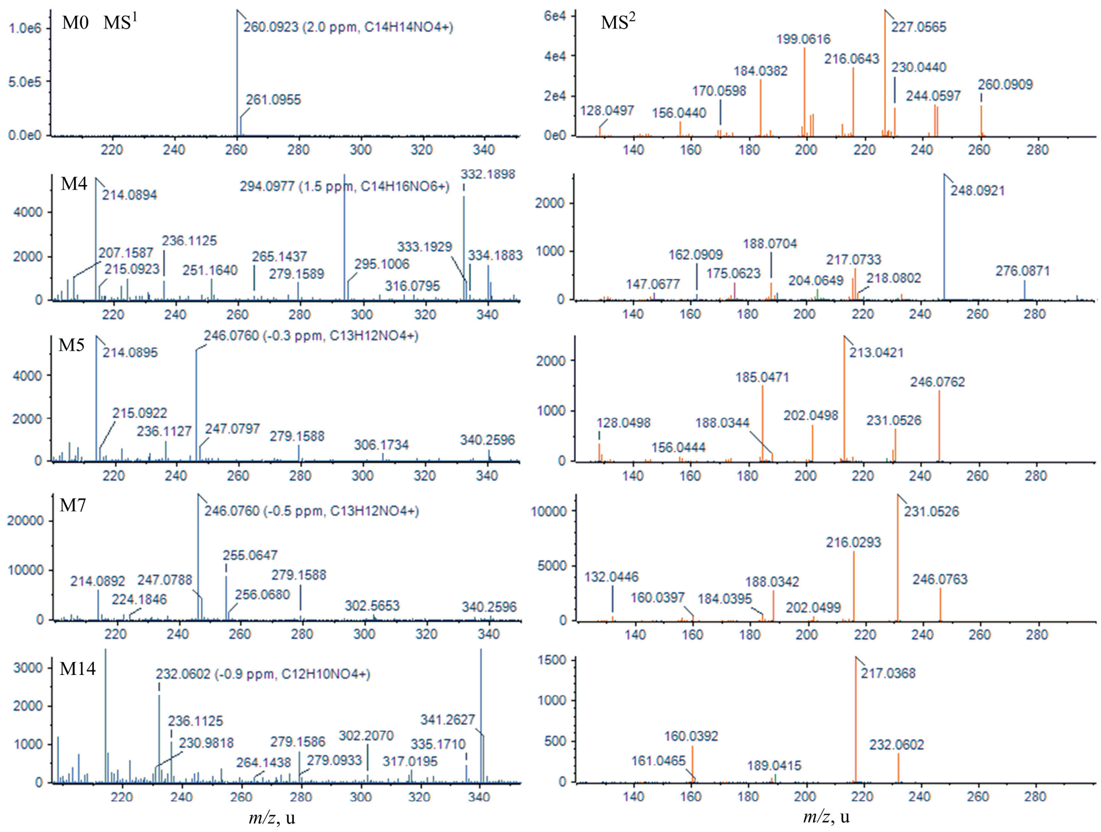

Figure S4. MS[1] and MS[2]spectra of parental drug and its metabolites identified in rat feces samples.
